# Participation of women and children in hunting activities in Sierra Leone and implications for control of zoonotic infections

**DOI:** 10.1371/journal.pntd.0005699

**Published:** 2017-07-27

**Authors:** Jesse Bonwitt, Martin Kandeh, Michael Dawson, Rashid Ansumana, Foday Sahr, Ann H. Kelly, Hannah Brown

**Affiliations:** 1 Department of Anthropology, University of Durham, Durham, United Kingdom; 2 Department of Social Sciences, Njala University, Bo, Sierra Leone; 3 Mercy Hospital Research Laboratory, Bo, Sierra Leone; 4 Department of Microbiology, College of Medicine and Allied Health Sciences, University of Sierra Leone, Freetown, Sierra Leone; 5 Department of Global Health and Social Medicine, King’s College London, London, United Kingdom; Baylor College of Medicine, UNITED STATES

## Abstract

The emergence of infectious diseases of zoonotic origin highlights the need to understand social practices at the animal-human interface. This study provides a qualitative account of interactions between humans and wild animals in predominantly Mende villages of southern Sierra Leone. We conducted fieldwork over 4 months including participant and direct observations, semi-structured interviews (*n* = 47), spontaneously occurring focus group discussions (*n* = 12), school essays and informal interviews to describe behaviours that may serve as pathways for zoonotic infection. In this region, hunting is the primary form of contact with wild animals. We describe how these interactions are shaped by socio-cultural contexts, including opportunities to access economic resources and by social obligations and constraints. Our research suggests that the potential for exposure to zoonotic pathogens is more widely distributed across different age, gender and social groups than previously appreciated. We highlight the role of children in hunting, an age group that has previously not been discussed in the context of hunting. The breadth of the "at risk" population forces reconsideration of how we conceptualize, trace and monitor pathogen exposure.

## Author summary

Studying how and why humans interact with animals is important to understand the transmission of zoonotic diseases (infectious diseases transmitted from animals to humans) and how to prevent and control them. We conducted a qualitative study to understand how and why people come into contact with wild animals in the Southern province of Sierra Leone, a region with numerous wildlife species known to carry zoonotic diseases. Previous studies on hunting in sub-Saharan Africa principally describe adult men as hunters and adult women as retailers of meat from wild animals. Based on our results, we seek to broaden the category of people deemed “at risk” of zoonotic diseases through hunting by including women and children. In particular, because of their limited physical abilities and social position, children hunt under different circumstances than those of adults. Our results have implications for zoonotic disease research and prevention, for example by ensuring children are integrated in health interventions and that their unique reasons to hunt are taken into account during such processes.

## Introduction

Recent occurrences of infectious disease outbreaks involving pathogens such as Lassa virus, Ebola virus and simian retroviruses have led to increasing concern about emerging zoonoses [1]. The probability of a zoonotic infection depends in part upon the frequency and nature of contact between animal hosts and humans [2]. Thus, in addition to the biological aspects of pathogen transmission, zoonotic diseases must be understood as resulting from social processes. Social science approaches are therefore an essential component in the study of infectious diseases [3]. More specifically, the environmental, social, cultural and economic aspects of animal-human interactions must be studied alongside human and animal behaviours to determine pathways for infections [2].

As a socially dense, gendered and sometimes secretive activity, hunting is a prime topic for in-depth social scientific analysis. Hunting and butchering wild animals poses a significant risk for transmission because such activities expose humans to animal secretion and fluids through bites, scratches and handling organs [4]. Outbreaks of Ebola Virus Disease (EVD), for instance, have been directly attributed to handling various wild mammal species during hunting or as carrion [5]. Hunting has been a major topic in disciplines such as anthropology, including in West and Central Africa [6–8]. However, the public health dimensions of these animal-human interactions are only beginning to be subject of sustained ethnographic consideration [9–12].

The multifaceted nature of animal-human interactions can pose considerable methodological challenges for research, particularly when such practices are hidden or secretive. Hunting, for instance, can be forbidden by law or custom; it can be associated with disease; and it can involve practices or knowledge that amplify social status or satisfy social requirements. The 2013–2016 EVD outbreak in West Africa heightened these ambiguities following a ban on hunting, sale and consumption of meat from wild animals. Using questionnaire surveys to investigate sensitive topics may introduce systemic bias [13–15]. In particular, children are more difficult to study through quantitative survey techniques and their role as a potential group at risk from zoonotic infection remains largely unrecognized. Such difficulties can be alleviated by immersive qualitative and open-ended study based on building trusting relationships, developed over lengthy periods of time. Long-term qualitative studies allow researchers to build a rapport with informants that can reveal information not accessible through other methods. Open-ended approaches, with a strong observational component, facilitate understanding of behaviours at the animal-human interface that are routinized and/or controversial [16].

Anthropological studies of animal-human interactions such as hunting and butchering practices can offer a critical entry point to understanding zoonotic risk dynamics [17, 18]. Ethnographic approaches help to frame public health understanding of the ways different social groups engage with animals and can inform the design of disease surveillance measures. Understanding the drivers of animal-human interactions is important when designing risk prevention strategies. Further, a fuller appreciation of such interactions can help to contextualize research in zoonotic disease ecology. This is of particular use in West Africa following the renewed interest in zoonotic disease ecology in the region with the presence of numerous wild animal reservoirs for zoonotic pathogens, including Lassa virus [19] and ebolavirus, which has possibly been circulating in West Africa for decades [20–22].

The aim of this study was to provide a finely grained description of human actors and behaviours that may serve as pathways for zoonotic infection from wild animals, and to understand the drivers behind these behaviours.

## Materials and methods

### Study site

**Fig 1:**
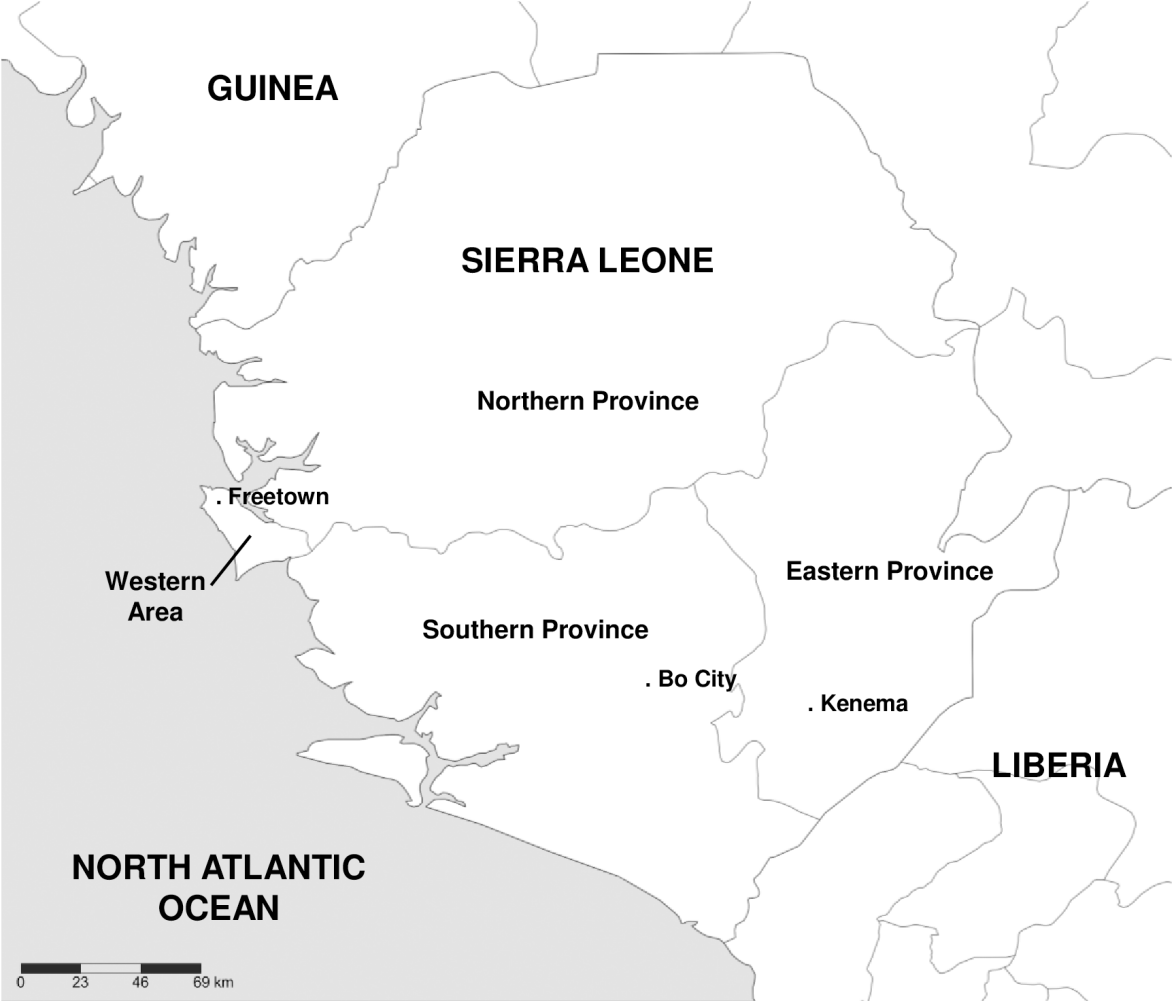
Map of Sierra Leone. Fieldwork was conducted in the Southern and Eastern Province, up to 50km from Bo City and Kenema, the two largest cities of these provinces (created with Simplemappr, www.simplemappr.net).

The fieldwork was conducted in the Southern (Bo, Pujehun and Moyamba districts) and Eastern (Kenema district) Province of Sierra Leone (**Fig 1**). We conducted fieldwork in urban and rural locations. Bo City is the second largest city of Sierra Leone and its inhabitants are involved in a range of economic activities including small-scale trading and salaried employment. Bo City borders swamps and grasslands merging into a mosaic of swidden farmland and secondary forests. In rural locations, three villages were chosen based on previous fieldwork and familiarity with the field researchers. These villages (between 6 and 12km from the outskirts of Bo City) were visited at a minimum twice weekly during the fieldwork and provided the core of the data collected. Six other villages, identified through snowball sampling, were chosen to represent more isolated areas (up to 40–50km from a major town) but were only visited between 1 and 4 times. Villagers depend on fishing, hunting, swidden farming, cultivation of small plots and small-scale trade for subsistence and income.

Fieldwork was conducted for a total of 4 months in 2015 (August, September, November, and December), overlapping the rainy (May–October) and dry seasons (October–May). We also draw on interviews and observations collected in May and June 2014 from the same study site. Some of the fieldwork took place during the EVD outbreak, although we worked in districts without active cases during fieldwork. Given the sensitive nature of our research during the EVD epidemic, we began by visiting informants known to us through previous fieldwork. Transects through villages, forests and swidden served to identify people engaging in behaviours of interest.

### Interview and discussions

We conducted semi-structured interviews and informal discussions until data saturation was achieved. The discussions were conducted in English, Mende or Krio (creole English). Interviews lasted between 30 and 60 minutes. The questions were pre-determined and covered food security, local gastronomy and forms of interactions with wild animals, which included their practical and symbolic significance. A separate question set covered the impact of the EVD epidemic and is discussed elsewhere. Photos of wild animal species were used to determine vernacular names and ensure accuracy of translation. Although the interview guides were pre-defined, questions were posed in an informal manner to encourage discussion.

### Observations guides

The observation guides used for direct and participatory observations covered forms of direct and indirect contact between humans and wild animals. Participatory observations were mainly done with trusted informants. Informants were given the opportunity to ask questions about the link between wild animals and EVD. Our answers covered risk factors for zoonotic infection and current hunting regulations. Thereafter, no attempt was made to challenge the activities observed, except to encourage basic biosecurity measures when handling animal carcasses.

### Written essays

We set two simple written essay questions for children aged 14 to 16 years attending the school of village A. The questions asked children to describe an animal that lives in the bush and to describe the last hunt in which they participated.

### Data processing and analytical strategy

Recordings and field notes were immediately transcribed into English by the field researchers (JB and MK) using Word 2011 (Microsoft Corp., Redmond, WA). The data was rendered anonymous from the onset and shared online with the research team. Analysis was carried out continuously and interview and observation guides were amended iteratively. Triangulation was obtained with three field researchers (JB, MK and MD) and multiple methods of data collection. Coding was done in MS Word 2011 using a thematic analysis. *A priori* codes included forms of interactions with wild animals, use of wild animals and food security. Inductive codes were applied to understand the social, cultural and economic context of these interactions.

### Ethics

The study was approved by the ethics committee of the Government of Sierra Leone and the University of Exeter. Participants were provided with information sheets that were read out. We emphasized that participants did not have to answer questions and could end their participation at any time without consequences. Written and oral consent was obtained from the respondent or a parent for participants under 18 years.

## Results

We conducted 47 semi-structured interviews and 12 spontaneously occurring focus group discussions, collected 13 essays and performed 14 days of participatory observations. Direct observations and informal discussions were conducted throughout. Informants included village chiefs, elders, teachers, housewives, farmers, small-scale traders and children (from the age of 5). Three hunters from one village refused to be interviewed. Among the respondents of the semi-structured interviews, one informant was interviewed twice. Interviews with 18 respondents were not recorded because they refused, or indicated that they preferred not to be recorded. Respondents were predominantly Mende (*n* = 42%) or mixed Mende (*n* = 7, 15%) and either Muslim (*n* = 18%), Christian (*n* = 22, 48%) or unknown (*n* = 6, 13%). There were 32 (70%) men and 14 (30%) women. Information about children was mostly collected during participatory and direct observations and informal interviews.

### Classification of animals

The Mende classify animals into three broad categories: livestock, pets (dogs and cats) and wild animals (“bush animals”). The term “bush animals” refers to species that live in or outside of villages but are not domesticated. Villagers discuss these primarily as a crop pest (e.g. rodents) or as resource to be exploited. “Bush animals” have individual vernacular names in Mende. In the following text, we group species according to their size, ranging from small (small rodents, squirrel, mongoose, bat, bird, amphibians, reptiles), medium (Gambian pouched rat, cane rat, brush tailed porcupine, genet cat, small non-human primates), and large species (forest antelope and forest hog).

### Trapping and hunting

We set out with about 7 adult hunters and a dozen children (aged around 6–12), most of whom carried nets on their head. Everyone brought their own cutlass (I brought one to fit in,) and dogs obediently followed their owners’ steps. You could tell that both dogs and people were excited by the hunt, and as we made our way through the bush, everyone became progressively quieter.

*The first hunt was unsuccessful and we moved to a second area (about a kilometer from the previous one)*, *again unsuccessful*. *To get there*, *we passed along a long fence with many traps set along it*. *The third hunt was successful; a grass cutter (cane rat) got tangled in the net and was jumped upon by the hunters*. *They kept it alive until I got there and then killed it by punching his head in (you cannot used a cutlass as it destroys the net)*. *Relatively fast to unconsciousness*, *no more than 7 seconds*. *Blood everywhere*. *The kill was immediately handed to a boy (who was very proud of it) and ran away in the bush with it on his shoulders*. *The hunt continued again and we moved twice more until the hunt was declared over* (field notes from a communal hunt DO-04A).

#### Techniques

**Fig 2:**
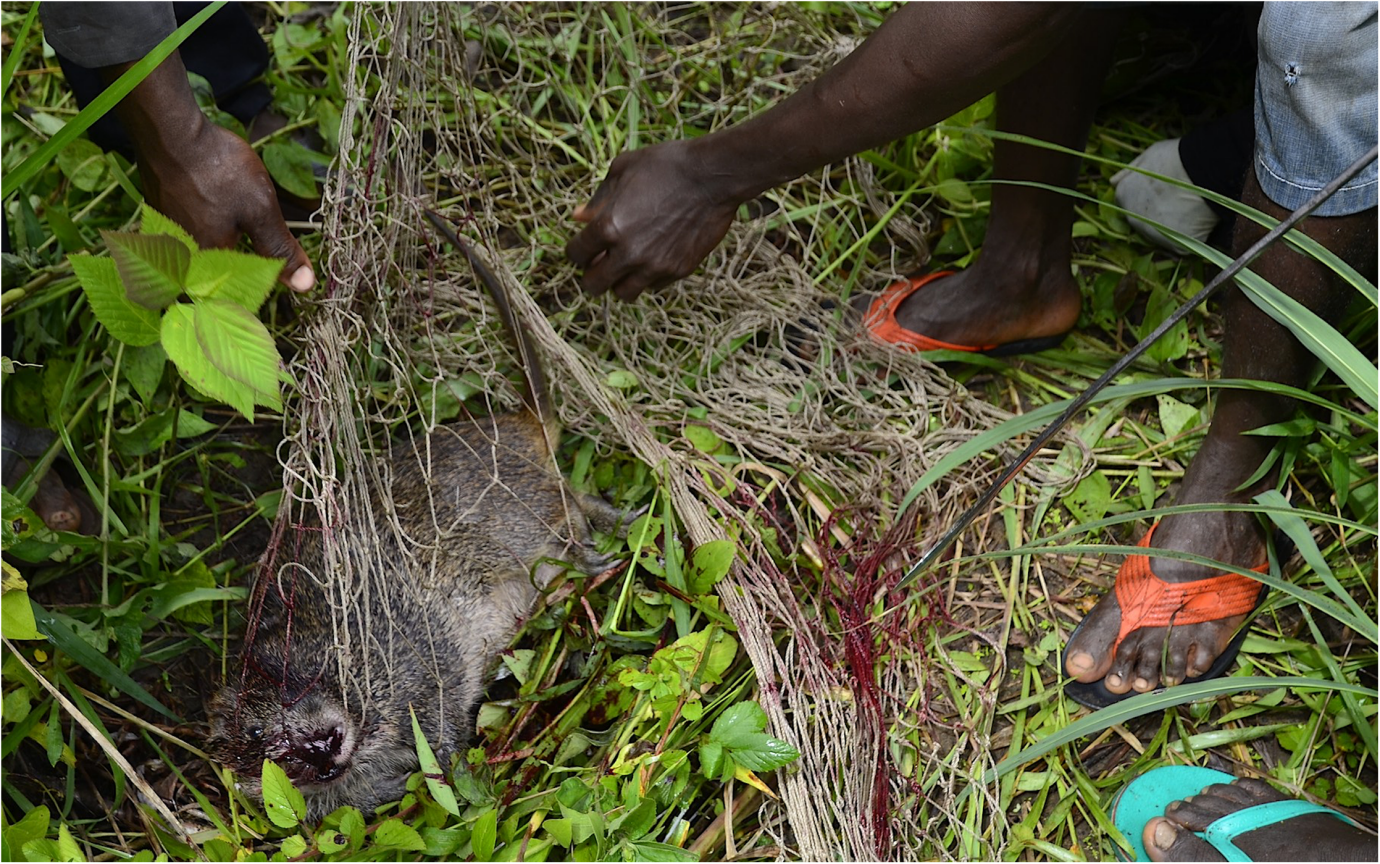
Communal hunting with nets. A cane rat (*Thryonomys swinderianus*) caught during a communal hunt. The animal was bludgeoned to death by hitting the skull with bare fists, rather than a machete, to avoid damaging the hunting net.

Communal hunting with nets is done with a group of people including net owners who encircle a delimitated area with their nets, and dog owners, who use their dogs to flush prey towards the nets. Other participants close off the rest of the delimited portion and flush out animals. During the communal hunt that we participated in, animals were bludgeoned to death with fists to avoid damaging expensive nets (**Fig 2**). Ideally, hunting excursions last until sufficient quantities of game are caught so as to share meat with every member of the party. For example, we participated in two hunts where a large cane rat was caught, in both cases, hunting activities continued for more than half of the day in the hope to secure additional game. Communal hunting parties are formed to protect crops by flushing animals but are rarely done because of the amount of coordination they involve. We only observed four episodes of communal hunting in two of our main study villages. According to elder informants, communal hunting was more common before the civil war (1991–2002) when meat was given to visiting dignitaries such as politicians, census officials, and tax collectors. However, the same informants affirmed that the custom of gifting meat to dignitaries was no longer practiced. They explained this by a shift in perception brought on by campaigns from non-governmental organizations following the civil war. These campaigns advocated for “democratic” values by reducing the servitude of village subjects towards the village chief and, by extension, to visiting officials. Whilst communal hunting was a feature of the village calendar, most hunting was carried out independently. In our study area, trapping (use of snares) is the most common method for catching animals. Different traps are adapted to target certain species, although some are relatively indiscriminate in the species they catch. Traps are easy to learn and build, but their upkeep can be time consuming because of the need for regular checking and repair. This, and access to snare cables, is the principal limitation to the number of traps that an individual will lay and there is considerable variation amongst individuals. We counted between five to hundred traps per owner, the latter which can take up to half a day to check.

Hunting with guns is frequent, but done in secret because firearm ownership has been prohibited since the end of the civil war. There is tacit knowledge in villages of who owns a hunting firearm. These are locally made barrel guns that use standard shotgun cartridges. Hunting is usually done at night with torches to startle and freeze prey, which are usually medium and large sized species. Army and police officers can legally shoot animals that cause severe agricultural damages (such as buffalos), but they are reported to use their privileges to hunt other species in collaboration with villagers who serve as guides in exchange for a share of meat.

Hunting dogs are trained to point, chase and kill small and medium sized animals. Other methods of hunting involves smoking prey out from burrows, use of slings and catapults (for small rodents, birds and bats), encircling patches of bush and cutting it down (“brushing”) or setting fire to it (for most species). Whenever an animal is spotted and the chances are deemed high enough to catch it, any method is used including bare hands, machetes and sticks. Running after prey until exhaustion was described twice, for a forest antelope and a cane rat and incidents of drowning exhausted animals were also recorded.

Bats are hunted with specialized methods. Cave dwelling bats (*pan devi*) are whipped with long sticks as they fly out from the mouth of caves. In our study area, access to and around certain caves is strictly regulated because they are used for male secret society (*poro*) ceremonies, thus non-initiates and women are forbidden to approach them. Prior to the ban on firearms, shotgun cartridges filled with grit were used to kill tree roosting fruit bats (*taje*) colonies. Since then, other methods are employed such as slings, catapults (a variant of a sling with multiple shots) and one village reportedly used a net strung between trees. The bad smell of insectivorous bats (“thatch bats”, *jassahun devi*) precludes them from consumption, but children will catch them from the thatch of houses and use them as playthings. Overall, bats are considered “*too strenuous to go after”* (informal discussion, town elder) and so are mostly hunted opportunistically, for example if they are roosting in small trees. However, one village that we visited was located near caves housing large bat colonies and villagers from surrounding villages assembled annually to hunt bats in late November. In these instances, villagers reported filling up bags containing up to 50 bats.

#### Skill acquisition and success rates

Hunting and trapping skills are acquired through observation and participation with experienced hunters and trappers, usually members of the family. If the household head is not a hunter or trapper, it is unlikely that other family members under his direct care will be either. In this case, hunting/trapping can be learned from friends, extended family, or through people employed to set traps on farmland. Children learn from adults and from each other. The success rates of trapping and other forms of hunting are inconsistent. One farmer described how animals *“can enter into the nets and still escape*, *so it is a game of luck”* (farmer, IDI-04A). One trapper responsible for about 60 traps caught animals only every few weeks, communal hunting rarely resulted in more than a few animals caught in a day. Communal hunting was considered more efficient than trapping, until rarefication of game made it less so.

#### Participation in hunting

Hunting with guns is the exclusive domain of males, and historically that of *kamajors*, loosely defined as “traditional hunters” [23]. *Kamajors* are distinguished by their membership to hunting brotherhoods, which, historically, requires months long initiation. They are respected for their hunting skills, their fearlessness in killing large, dangerous animals such as forest hogs, buffalos and leopards (now locally extinct), their knowledge of medicinal plants and their historical role in protecting villages from wild animals and enemies. This status is slowly being eroded and replaced by hunters that use guns but have not gone though the initiation necessary to enter hunting brotherhoods. Despite this, such hunters are respected for their knowledge of the forest and their ability to navigate it at night, because doing so places them in contact with the world of witchcraft and sorcerers. Because witches and sorcerers are considered to have the ability to navigate between human and animal forms, large game hunters will cut the tail off large nocturnal animals as proof of having killed an animal rather than a human. Whether they hunt with guns or not, men, including traditional hunters, participate in all types of hunting.

**Fig 3:**
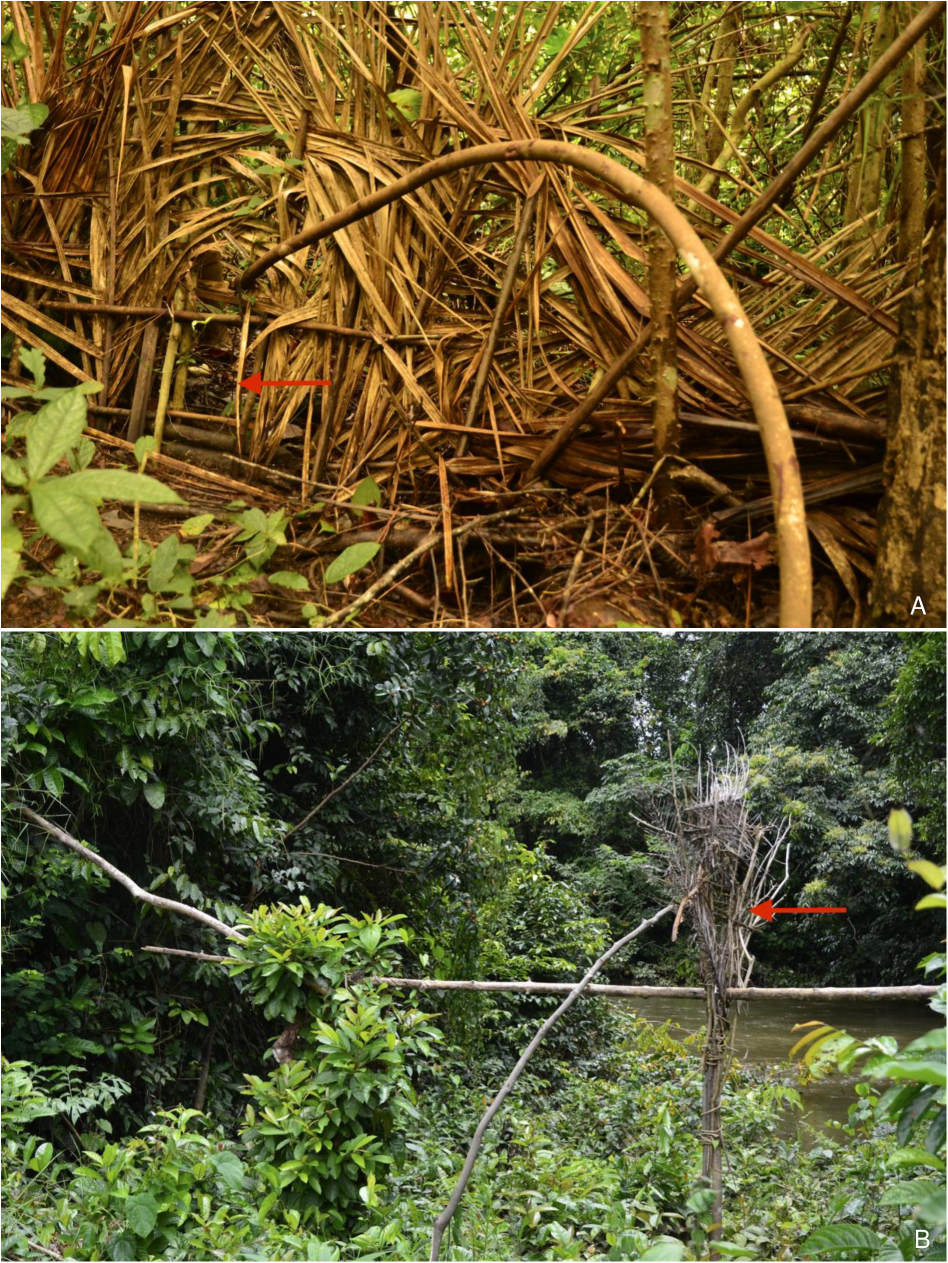
Snare traps. A common trap (*dahin*), which can catch most species of mammals and reptiles (A) and a trap specifically designed to snare small non-human primates (B) as they cross a cleared portion of forest on a branch. These latter traps are uncommon because they are difficult to build and non-human primates learn to avoid them. Both traps work by snaring animals with use of a spring mechanisms when they pass through a sensitive trigger mechanism (arrows).

Women participate in communal hunting, helping to flush prey into nets. They are also opportunistic hunters when working in the fields or during other activities. Many women recounted an episode where they caught various species of animals, typically during fishing. Two accounts by women described drowning a deer and large snake during fishing. Women will recount such episodes with pride at proving themselves to men, and happiness at having contributed meat to the household. Women do not generally hunt with traps mainly because of the danger posed by the powerful spring mechanisms used (**Fig 3A and 3B**). However some women do routinely engage in trapping (and in other male activities such as palm oil harvesting) typically because they do not have a strong family support structure, such as widows without children. In general, discussing women who hunted or trapped did not generate any negative comments when discussed with men. However, women who surpassed men in these activities were forbidden to do so, as they were considered to breach traditional gender roles.

**Fig 4:**
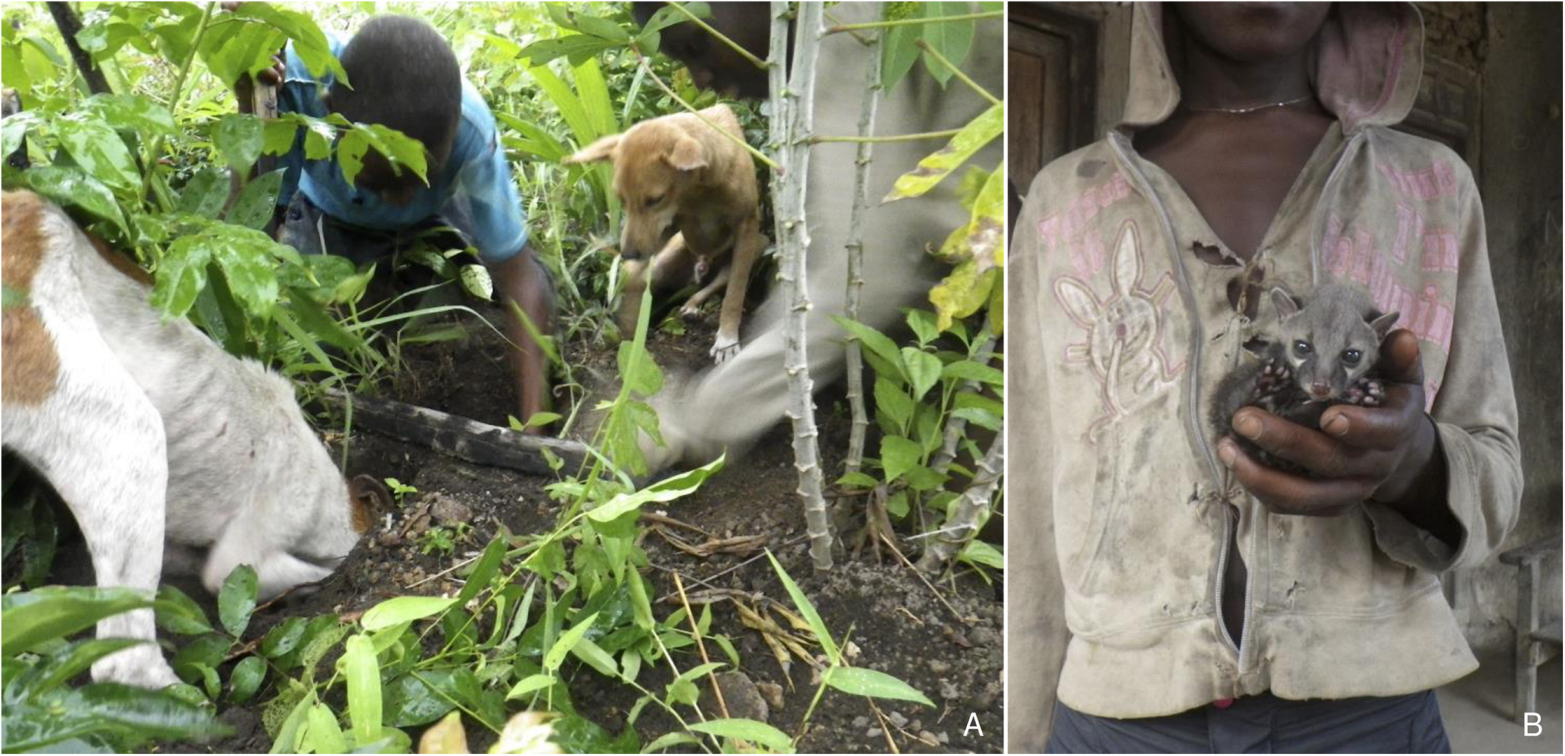
Children hunting. Children hunting with dogs owned by the family and borrowed from an unknowing neighbour (A). The dogs detect or chase rodents into burrows, which are then dug up by the children. A genet kitten (*Genetta* sp.) (B) The kittens were reared by children in a chicken coop and eventually eaten.

Hunting amongst children parallels that in adults, with boys more likely to hunt than girls. Boys hunt alone or in groups, starting from about 7 years of age. All forms of hunting and trapping are practiced (with the exception of gun hunting). However, owing to their limited physical ability, children use smaller trap constructions and target smaller species (**Fig 4A**): “S*ometimes we dig*. *We used to search for their* [squirrel] *holes*. *Yes*, *where they entered*. *Even where we mark one hole we search*, *one person will stop there and trace it where is stops* [exits]. *Another person stands there and will begin to dig (laugh); we come close to meat” (*young man, IDI-08A). One teacher described the opportunistic hunting conducted by children: *“I just see three children with 2 kpomie* [tree hyrax]. *I said how did you catch these things*? *He said they are just on their way going to their farms and they saw them on the road*, *they were about to cross the road*. *So they kill them on the road*. *Because the kpomie is not able to run like sewei* [cane rat]” (IDI-09A)”. One child with a reputation for being a good hunter (but poor student) boasted of owning at least fifteen traps. When baby animals are caught, they are reared to adulthood as pets and then used for food, usually under the responsibility of children (**Fig 4B**). We frequently observed children playing with wild animals even beyond the point of death, with particular fascination for inspecting and opening mouths, and stroking fur. In Bo City and villages, boys hunt cats and other animals in groups and attach value in cooking them amongst each other, which is known as “*boys cooking*”.

#### Catch distribution

The kill is divided between the participants according to traditional custom with specific body parts distributed to net and dog owners and the person that caught the animal. The help provided by women is appreciated and they are entitled to equal portions.

The circumstance of the kill and the size of the animal determine how an animal is shared between members of the family and who will butcher it. If an adult household member judges that his or her catch is too small to suffice for the household, he or she may decide to keep it for him or herself only: “*if it small*, *as I cannot share with my wife*, *I eat it alone*” (farmer, IDI1-17). This scenario is particularly common when farmers catch a small animal while farming and have access to cooking utensils in their farmhouse. Larger game is bought home and usually sold or cooked by women.

There exists a tacit requirement to share a part of the meat, if sufficiently large, with the wider family or close friends if they reside nearby. Refusal to share creates a negative reputation of “greediness”, which sometimes pushes villagers to hide and sell meat outside of their own village so that they are not pressured into making too many gifts. Sharing is also expected in certain circumstances, for example a wild animal caught on someone else’s land or with someone else’s dog.

#### Temporal characteristics of hunting and trapping

Hunting and trapping occur all year round but predominantly during rice farming (planting to harvest: April–December) and the rainy season (May–October), when swidden rice nears maturity and is particularly vulnerable to crop pests. During this period, fences with traps are built around fields. In their school essays, children reported hunting daily or weekly, which was corroborated with field observations. The essays were set just before the rice harvest when grain stocks from the previous year are at their lowest (the “hungry season”) and the requirement for other sources of food (e.g. animal protein) is high.

#### Spatial characteristics of hunting and trapping

We observed hunting, trapping and fishing in rural, urban and peri-urban areas of Bo City, where the urban landscape merges into agricultural land (swidden), swamps and tertiary forests.

### Food preparation and consumption

“Y*ou know monkey does not have too much flesh, when the pepper, the maggi* [spices], *goes into the bone you will suck and enjoy it*” (student, IDI-09B).

**Fig 5:**
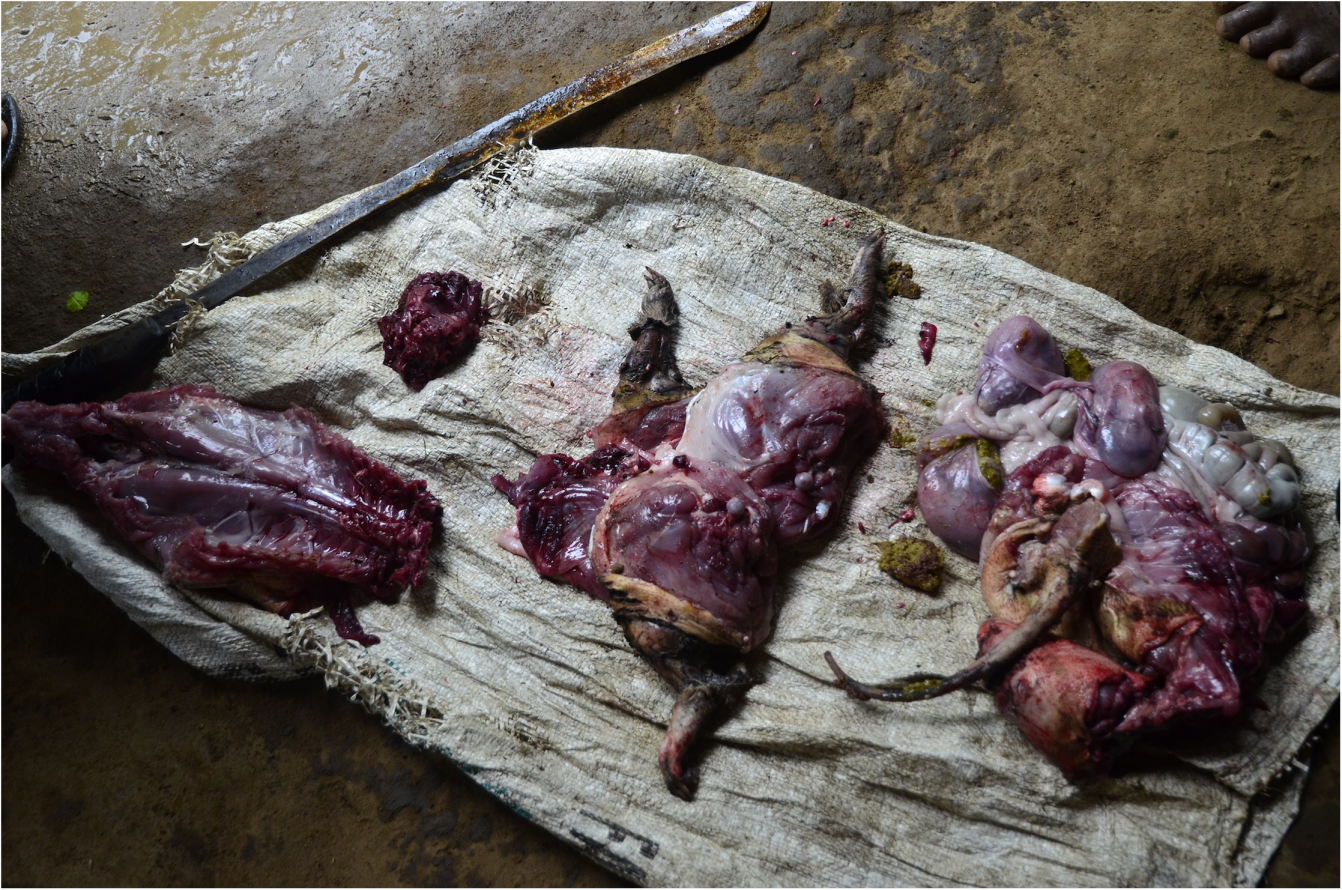
Butchered meat following a communal hunt. A cane rat (*Thryonomys swinderianus*) caught during a communal hunt. The carcass is singed, butchered and shared between the participants who either cook it together or bring it back to their household.

The only preparation methods for meat that we observed involved singing the hair, followed by gutting and butchering (**Fig 5**). When selling meat to traders, hunters usually sell the entire carcass because of the higher price it will receive. In this instance, the market seller butchers the carcass. Only certain parts of animals are not eaten (nails, hooves, and horns). The gut and the gall bladder are the only viscera that are consistently removed, as they are deemed to taste bad and be poisonous. Some people choose to remove the genital organs because of the smell. Any unwanted organs are thrown away, fed to dogs, or kept for use as bait in fish and crustacean traps. Bones are eaten entirely unless too big, in which case they are broken and the marrow is sucked out, which is particularly prized by some.

Preparation is not gender specific. Cooking is done over a fire. It involves grilling meat over the fire, frying it in palm oil, boiling it in water, or more often, a combination of all. Leftover meat can be conserved by smoking it over a fire. Palm oil brought to boiling point is widely believed to kill pathogens and other impurities in food, such as rodent poison, which is occasionally used to protect swidden. While the majority of people prefer eating well-cooked meat and gag at the suggestion of eating raw meat, two people stated that they prepared a soup from boiled meat that retains raw blood, as it is considered more “*nourishing”* in terms of protein, as well as better tasting: *“*[*We*] *let the blood just escape a little and then (laughs) we start eating it* [*the meat*]. *Sometimes we do not cook it*, *we do not cook*, *we only put it on the fire*, *make it look fine then we eat it*. *And then you will really eat and enjoy it”* (urban farmer, IDI-05B).

During our observations, carcasses were always handled with bare hands and blood was rinsed off with water or sometimes with chlorine water, which is present in villages since the EVD outbreak.

### Reasons for hunting

*“We hunt [deer] because we need money and meat and use the skin to make drum”* (middle school student, essay on hunting).

#### Food

A major reason for killing wild animals is for an immediate source of protein. This needs to be understood in the context of available alternatives: cows are rare in the Southern Province, their meat it expensive and only available at market points. Goats and sheep are more common but are not routinely eaten as they serve for rapid cash income (e.g. funerals and weddings). Chicken and ducks are used for rapid cash income, gifts, or for occasional personal consumption. The most common and cheapest source of protein is commercial fish (smoked fish and frozen fish, which is delivered to even the most remote villages) and forest products (invertebrates and freshwater fish), which are inexpensive or free, but seasonal.

The importance of eating meat is related to cultural notions of what constitutes a flavoursome and healthy diet. Rice is the staple food and should always be accompanied with a meat “sauce” (*ndahain*). The negative formulation of “*empty rice*” refers to rice that does not contain sauce, or contains a sauce without meat (definitions vary, but the absence of meat is the defining feature). “*Empty rice*” is never a voluntary choice and there is a daily pressure to provide some form of meat within a household. Frozen fish does not fulfil this demand as it is considered to lack “*taste and vitamin*” (farmer, informal interview 05A). In contrast, red meat is discussed in terms of health benefits, with vocabulary borrowed from nutrition awareness campaigns that recommend giving meat to malnourished and anaemic children. Red meats provide “*sound health*” (farmer, IDI-25A), “*energy”* (farmer, IDI-03A), gives “*blood faster*” (farmer, IDI-30A), “*makes body and mind strong*” (farmer, II-05A), and is “*more nutritious than ice* [frozen] *fish*” (farmer, IDI-03A). Further, fish does not provide the same sense of satisfaction and satiety that red meat does in an otherwise bland and repetitive culinary environment. Hence even though small mammal species provide little meat, they give a much-appreciated taste to the *ndahain*. Small species are also relatively abundant and easy to catch: *“we go to the swamp and brush [a form of hunting] so easily we can get meat*. *And that is the simple way we can get the meat faster”* (teacher, IDI1-21). So important is the need for protein that our principal informants reported that it is difficult to pass the opportunity to scavenge an animal found sick or dead: “*the dead animal I found (…)*. *It was freshly dead*, *like a snake bite that* [bit the] *sewei* [cane rat] *because normally if it is being shot by a gun we can smell the cartridge scent on the animal but this was not the case*. *I wanted to believe that it was a snake that bite that sewei and we found it on our rice farms*, *so because it was fresh we had to eat it”* (IDI-09A, village teacher, pastor and farmer). Even during the EVD outbreak, one housewife stated: *“even now*, *when I see one [an animal]*, *I will make use of it*, *even a dead one”* (IDI-21A).

Although the driving force behind communal hunting is crop protection, people will avoid wasting meat whenever it is made available, as occurs if an animal is caught, which is regarded as a blessing. Revelatory of the importance of not wasting animal protein is the “*pepper law*”—a punishment given to anyone who, through carelessness, lets game escape during communal hunting. Eating (chilli) peppers and other punishments, such as the obligation to clear the village of undergrowth, pay a fine or not receive a share of meat, point to the importance of not letting game escape. Providing an insight into the sensations felt during hunting, one farmer described the pepper law as “*painful* […] *so next time when I went to take the hunting net I was ready for the animal*, *because for the first time when I had never caught an animal*, *when I saw the animal coming*, *my entire body was trembling*, *so it made me leave it to go*, *so I chew that pepper*. *So next time when we went I challenged it* [the animal], *I said I was going there again that time I did not chew pepper again when the animal came*, *that day I caught two fritambos* [duiker antelopes]*”* (IDI-05B).

#### Crop protection

Protecting crops from pest animals is a key preoccupation of farmers. It involves erecting wooden fences with traps around swidden and building watchtowers to kill or ward off animals. Undertaking communal hunts to flush animals away is considered an important, and sometimes obligatory, practical duty. Farmers appreciate when hunters operate in vicinity of their farmland in the hope that it will flush pests away and spare their crops: “*I have made effort to drive away those animals so that they will not destroy them* [crops] *again*. *So that is why people do tell me thanks*. *It is for that reason that I am very popular in this area*, *I do help so many people”* (urban farmer, IDI-11B).

#### Income

Animal protein is a product in high demand that can be easily sold and guarantees rapid cash flow. The decision of whether to keep wild meat for personal consumption or for sale varies between individuals and situations. The decision making process involves implicit cost-benefit calculations taking into account the potential revenue of the animal, the amount of money saved by not buying fish, and appetite. In general, small species with little market value (bats, squirrels, other small rodents) are kept for personal consumption or, if sold, usually only within the village. Bats are considered too small to provide much meat but are sufficient for the *ndahain*. For this reason they are mostly given to friends and family members, including those that have emigrated to urban areas. They can be sold within villages at a low price (U$ 0.1–0.5). Medium sized species can be divided for personal consumption and sale in villages or towns. Large game is sold either in town where urban residents will pay a higher price for it, or in villages if the seller is assured that there are enough clients. Gun hunters most frequently engage in commercial trading, as they are more likely to kill large game and need to recover their investment in cartridges. They have established networks of middlemen and retailers and can invest more time in hunting and trapping. For small market chains (intra and inter-village trade and trade with Bo City), meat is most commonly sold fresh, either as entire carcasses or in butchered pieces.

#### Other uses of animal products

We documented the use of animal species for medical purposes. This includes toads (for whooping cough), raw monkey skin or burnt squirrel hair (wrapped around burn wounds), snakeskin and intestine of the brush-tailed rat (to ease stomach pain), and duiker horns made into necklaces (for babies with convulsions or other ailments). In urban areas, monkey pepper soup is a popular dish classically eaten between friends on a night out because it is thought to lessen the effects of hangovers. Hides from forest antelopes are still occasionally used to make drums and farming gloves and snakeskins can be made into belts.

#### Social importance of wild meat

In rural areas, a boy who has never caught an animal is considered “idle” (lazy). The ability to bring back a wild animal is part of a set of skills that is required of an adult farmer. As one boy explained, “*I don't bring it* [rat] *to town because*, *if my parents see it they will frown at me that the only animal I can catch is a rat*, *that is why I eat it in the bush”* (FGD-16B). Boys and young men will bring meat back as an attempt to seduce girls, who will cook the meat in privacy and share with her suitor. This can be reciprocated when the girlfriend’s family obtains game; the girl will keep some and share it covertly with her boyfriend.

Meat plays an important role in ceremonies. It is, for instance, a traditional requirement at funerals. When families cannot afford domestic meat for funerals, villagers or friends will hunt with them to spare them the embarrassment of being unable to provide meat. Rarely, communal hunting is organized to provide meat for religious occasions. Hunting and trapping skills are a main feature in initiation rites of male secret societies for entering adulthood, but details are not discussed with non-initiates.

#### Taboos and religious interdicts

Many Mende believe that the characteristics of what an animal feeds on are transferred onto the person who eats that animal. Animals caught in proximity to graveyards, waste sites and latrines are usually discarded, although we did note occasional exceptions, especially with children. These are more likely to hunt and consume peri-domestic animals such as small rodents that are usually shunned by adults.

The only species consistently avoided, irrespective of religion, gender or age, are the musk shrew (widely believed to transmit Lassa fever), monitor lizards (its prominent forked tongue relates back to the concepts of twins and associated taboos), cobras (associated with untrustworthiness) and dogs (because of their practical importance in hunting and defence, and the emotional attachment many people have with them). Other taboos varied between individuals, including chimpanzees, tree hyraxes and namesake animals.

Not all Muslims adhered to the prohibitions laid out by the Quran (which identify animals such as swine, non-human primates and rodents as haram—forbidden) as poverty and hunger are considered an adequate justification to eat haram meat, so long as it is not made into a habit. Taboos only apply to consumption as all species can be killed, either because they are crop pests or feared. They are then thrown away, sold or given to people who do not share that taboo. As one farmer who did not eat monkey stated: “*I will not sell it because I have people* [who] *like monkey*, *I don’t eat it but I have people that eat*. *So if I catch that one*, *I will give it to them*” (IDI-29A).

#### Children

Children hunt for the same reasons as adults, but their hunting is shaped by different social obligations and physical constraints, which, crucially, determine the type of meat they obtain and the way it is prepared and eaten.

Catching wild animals forms part of the domestic responsibilities of children in a household. Parents describe sending their children out to hunt, either routinely or only in certain cases, for example when adults have not been able to secure protein for that day. Although not all parents consider it the responsibility of children to contribute directly to the *ndahain*, some took a strict approach and threatened unwilling children with “empty rice”, to “*encourage”* them to go next time. As one child summarized; “*if I am healthy and do not go hunting*, *my parents will shout at me*, *and if they buy meat when they cook*, *they won't dish for me”* (school child, FGD-16B).

Further, in Mende households, the hierarchy of the family members is mirrored in the distribution of food: first the household head, then wife or wives and finally, children in decreasing age. In the words of one child, it is the “*father* [who] *will decide what to give and it* [the meat] *is under his authority even though* [the child] *caught it*” (IDI-14A), hence “*here*, *the children get the bones*” (farmer, IDI-30A). One mother explained that: *“if my boys* […] *come home with meat and I prepare it*, *let’s say for instance a portion I cut it into 6 portions*. *I will bring two portions to my husband*. *Two portion for me*. *And the other two portion for the two boys*. *That's how I will distribute it”* (farmer’s wife, IDI-16A). Thus, children can be disinclined to bring home any meat they catch, preferring to roast their catch secretly in the forest to avoid sharing it and being punished by adults. Such considerations form the basis of a social gathering known as “boys’ cooking”, during which: “*you can eat a lot*, *the way you want to eat*. *But maybe at home you just put it out of your small basin* […], *it’s not even enough for you*. *But the boys' cook; you eat and reserve* [keep] *another one* [portion of meat]. *When you eat you will go to the field*, *play your ball*, *later when you come back you can continue to eat*” (IDI-20A). Children described how, after catching an animal in the forest, they pondered the costs and benefits of eating it alone or bringing it back to the household. Such calculation is based on the size of the catch and the daily circumstances. Indeed, a careful balance must be sought, as if their parents find out that their children ate wild meat on their own, they will punish them with “*empty rice*”, or as one mother succinctly explained: “*if he does not share we also won’t share*” (farmer’s wife, IDI-21A).

If children make the decision not too share any meat with their parents, they will try hard not to be caught. Consequently, children will quickly, and often incompletely, roast animals so as to speed up the process and avoid detection through smoke and smell. This covert behaviour also holds true with species where the parents have banned consumption (e.g. “town rats”). In urban and peri-urban areas, children will catch rats, lizards and birds and eat them in hiding and parents bemoan the difficulty of controlling them.

We observed children catching and selling fish, amphibians and rodents. One entrepreneurial child sold arthropods to a school lab and animals as pets (mongoose, herons, birds of prey, NHPs). The income is then given to the household head and can contribute directly to schooling. As one school child explained: “*I killed a* [brush tailed] *porcupine*, *last week; I sold mine because I needed lunch to come to school*” (FGD-16B). Such small-scale trade by children is sometimes undertaken in secret for pocket money.

## Discussion

### Hunting behaviours and risk for zoonotic infections

The most common activity placing humans in contact with wild animals observed in our study was hunting and slaughtering, which are associated with zoonotic disease infections and disease emergence [24]. Understanding how differences in demographic, socio-cultural and economic characteristics influence such activities is important to inform pathogen surveillance and prevention measures.

Our research suggests that the “conventional” narrative of hunting and its role in pathogen transmission is incomplete. Previous research on hunting in Western and Central Africa commonly describes an activity conducted by adult males, while butchering and trading wild meat is done by women who are exposed to fluids through cuts and scratches [25–27]. This narrative of the “cut hunter” attributes pathogen emergence to “bushmeat hunters” who are invariably assumed to be adult males [11]. In addition, children are rarely thought of as being in contact with wild animals despite being presumed index cases in at least three EVD outbreaks [28–30]. Further, to our knowledge, questionnaire surveys looking at exposure to wild animals do not recruit subjects below 15 years of age [25–27, 31], yet we frequently recorded hunting among children below this age group. One study on animal-human contacts in Uganda suggests that children from the age of 3 years are exposed to non-human primates, however these results were derived from adults responding on behalf of their children [32].

We previously showed that hunting of small rodents is more widely distributed across age, gender lines and social groups than previously appreciated [14]. In our present study, we sought to determine whether such observations were also pertinent to other species of wild animals, in particular those species that are not present in domestic spaces (as small rodents are) and might be associated with different hunting norms. While our research confirms that among the Mende, hunting is, indeed, considered a traditional adult male activity—the respective roles of hunting and fishing among men and women reflecting divisions of activities that mark gender identity [6, 8]—we find that children and women are significant actors in complex collaborative practices for catching and preparing wild animals. With the exception of large species that are deemed physically dangerous and are associated with witchcraft (buffalo, forest hogs, leopards), the participation of women and children does not conform to assumed gender and age-related roles. Rather, hunting, slaughter, consumption and trade of wild animals are determined by individual circumstances and practicalities. Crucially, contact with wild animals often involves children who, compounded by traditional family hierarchy related to food access, frequently engage in high-risk practices during hunting and preparing meat from wild animals. Thoroughly cooking meat is considered sufficient to inactivate EVD in blood, but consuming undercooked meat, which was reported by children and adults for different reasons, is likely to present a risk of infection [33] and a similar degree of risk may exist when consuming bone marrow.

### Hunting species and distribution

Not all species of wild animals present the same risk of transmitting zoonotic pathogens. For example, certain species of fruit bats are suspected reservoirs for ebolavirus [34] and although we did find some villages organizing bat hunts, we did not find any evidence of systematic bat trade. This could however be specific to ethnic groups or villages and requires further investigation.

Other species of mammals including duiker antelopes and NHPs are susceptible to ebolavirus [35, 36] and hunting sick animals or scavenging carrion is a major risk for ebolavirus infection [5]. We did not identify any particular taboos against eating species that are known to pose a risk for zoonotic diseases, or against collecting fresh carrion, however we did not consistently ask whether people would eat sick wild animals. The process of trapping does not allow trappers to monitor the health of animals before killing them. Further, raw meat is widely distributed across commercial and social networks, with the potential to spread pathogens, with limited possibility for monitoring or traceability. Species, and their associated pathogens, are distributed according to criteria related to market value. Many of the taxa associated with zoonotic pathogens, such as small rodents [37] and bats, have little market value, and are mostly kept for personal consumption and inter-village trade. Children privilege such small sized taxa for their ease of hunting and their low market value, an observation also reported in a nutritional survey of animal species consumed among children in the Democratic Republic of the Congo [38].

We documented occurrences of urban hunting in fringe sites of Bo City, which suggests that such anthropogenic ecotones should be targeted in disease prevention strategies. Although such zones have previously been associated with pathogen emergence [39], our findings stand in contrast to common intervention designs which assume, incorrectly, that there is little contact between humans and animals in urban zones, as has recently been described in Uganda [32].

### Incentives for hunting

Sierra Leone has one of the highest rates of malnutrition and child under-nutrition in the world [40]. In this context of chronic food insecurity, disposing of hunted or trapped game—an important source of nutriments for growth [38, 41]—is rarely an option, especially where access to alternative sources (fish or domestic animals) is scarce or expensive. Family hierarchies prioritise protein consumption among adults, which compounds the difficulty faced by children in obtaining animal protein, encouraging them to hunt. We previously reported how the consumption of rodents is strongly linked to food security [14] and extend this observation to other wild animals that are considered a threat to crops, on which the Mende are highly dependent. The link between crop protection and species hunted has been illustrated in the Eastern Province of Sierra Leone, where cacao farmers were observed to commonly eat monkeys (a cacao pest) hunted on their farms [6].

Adult informants also discussed wild meat in terms of taste, perceived therapeutic and nutritional value, and as a source of income generation, as previously reported in Western and Central Africa [42, 43].

### Changes in hunting patterns

Social, political and economic processes can influence host-pathogen dynamics, for example through changes in reservoir abundance and contact with reservoir hosts [44]. Comparing current practices with accounts from older informants, we described how social changes have modified interactions between humans and wild animals. Communal hunting was discouraged in post-civil war policies because it had been used as a means for village chiefs to impose their authority upon subjects and test for political dissent [8]. This coincided with an increase in fast reproducing, resilient species such as rodents that thrive in a modified agricultural landscape [45]. Previous studies have shown how changes in agricultural practices can influence biodiversity and lead to adaptions in hunting practices, for example “garden hunting” near domestic spaces [46] and trade of wild meat [47]. Such observations support our data that the increasingly small size of animals hunted no longer justify sacrificing time for communal hunting and could explain the reported increase in the use of traps and focus on trapping smaller species, with the potential for changes in zoonotic pathogen ecology. Post-war policies also directly influenced hunting practices by imposing a firearm ban, making bats more difficult to hunt in Sierra Leone compared to Guinea where shotguns are common, and cartridges are loaded with grit to kill large numbers of bats (Bonwitt, J.; pers. obs.).

### Field challenges and limitations

Our fieldwork was affected by the EVD epidemic. Sensitization messages erroneously emphasized the risk of infection through contact with wild animals and hunting was penalized. These measures raised the degree of respondent anxiety on the topic of hunting. The quality of discussions often considerably improved when we refrained from recording interviews.

For ethical concerns, the research team answered frequent questions about the risks of ebolavirus infection from wild animals, which arose during discussions and may have affected our results. Our presence initially generated suspicion; however this was minimized thanks to our work in the area prior to the epidemic. Through observations, discussions and participatory observations, we learned to discern the subtle traces of hunting and trapping activities, such as people with hunting nets or concealed rifles, concealed traps or a cleaned village (a punishment imposed for letting prey escape). Despite these reassurances, we cannot exclude the possibility that we underestimated the frequency of certain behaviours of interest or missed some altogether.

Although we describe behaviours occurring among women and children, the majority of our semi-structured interviews were conducted with males (70%). However, much of our data was obtained, and indeed strengthened from spending time in villages, conducting participatory observations and informal interviews with women and children. Our research could have benefited from more interviews with women, for which a female field researcher would have been beneficial.

Our study could have been enriched by quantitative data. However, we sought to address the paucity of qualitative data on hunting as explored from a public health perspective. In providing a finely grained description on hunting practices we hope that our results will broaden the scope of future quantitative research on this topic.

### Conclusion

Our observations corroborate previous studies of hunting throughout West and Central Africa [6, 11, 25–27, 31, 32] but emphasize the social nuances of the practice by expanding on the diversity of actors, social norms and motivations involved. The “cut hunter” narrative which assumes most hunters to be adult males has underpinned disease intervention strategies, and remains a subject of debate and research [48]. Previous research has shown the need to expand beyond the “bushmeat paradigm” to include other forms of animal-human contacts as risks for zoonotic infections and that are unrelated to hunting practices [32]. Yet even within the much studied “bushmeat paradigm”, we find that the diversity of actors hunting wild animals and the breadth of the "at risk" population forces reconsideration in how we conceptualize, trace and monitor pathogen exposure. These results also underscore the challenges of interventions, surveillance, research and sensitization campaigns. To address such complexity, intervention strategies should become more diversified and context-specific. In particular the role of children should be recognised; specific intervention strategies should be tailored to children’s specific hunting practices.

Finally, our findings provide a base for further investigations to determine risk factors for zoonotic infections in the West African region. A better understanding of the interactions between humans and reservoir hosts can help to elucidate the mechanisms of disease spillover into human populations in Sierra Leone [49] by linking epidemiological, ecological and ethnographic data.
