# Correction: Participation of women and children in hunting activities in Sierra Leone and implications for control of zoonotic infections

**DOI:** 10.1371/journal.pntd.0006476

**Published:** 2018-05-07

**Authors:** Jesse Bonwitt, Martin Kandeh, Michael Dawson, Rashid Ansumana, Foday Sahr, Ann H. Kelly, Hannah Brown

There is an error in the seventh sentence of the Results section. The correct sentence is: Respondents were predominantly Mende (n = 41, 89%), or mixed Mende (n = 4, 9%), or Kono (n = 1, 2%), and either Muslim (n = 18, 39%), Christian (n = 22, 48%) or of unknown religion (n = 6, 13%).

The Data Availability statement for this paper is incorrect. The correct statement is: The qualitative data from which this article draws is deposited in the UK Data Service ReShare repository https://dx.doi.org/10.5255/UKDA-SN-852917. Due to the emergency conditions under which research was conducted, and the incriminating nature of some of the information collected, transcripts are not open access but can be disclosed upon request to the authors if particular conditions are met.
